# It takes more than agency: Linking support from teaching staff, career engagement, and movement capital among university students

**DOI:** 10.3389/fpsyg.2022.1083698

**Published:** 2022-12-08

**Authors:** Gerardo Petruzziello, Audrey Ansay Antonio, Rita Chiesa, Marco Giovanni Mariani

**Affiliations:** Department of Psychology “Renzo Canestrari”, University of Bologna, Bologna, Italy

**Keywords:** career engagement, support from teaching staff, movement capital, university students, career self-management, online learning, career development, transition to work

## Abstract

**Introduction:**

Career self-management behaviours are key to overcoming the challenges of entry into the labour market. Combining the Conservation of Resources and Career Self-management theories, this study delves into the concept of Career Engagement among Italian University students by examining its relationship with Support from teaching Staff and Movement Capital. We hypothesised a mediation model in which Support from Teaching Staff predicts Career Engagement which, in turn, fosters Movement Capital. As the pandemic led to the adoption of online learning solutions, we also explored whether and how the interaction between teachers and students during online classes moderates the said mediation relationship.

**Methods and results:**

We collected data from 276 Italian University students through an online questionnaire. Results supported the mediation hypothesis, corroborating the mediating role of Career Engagement. We also observed that this relationship is stronger at medium and higher levels of online interaction between teachers and students.

**Discussion:**

Findings contribute to existing evidence about the role of Career Engagement in facilitating career resources’ acquisition and extend the understanding of its contextual antecedents in Higher Education contexts. Results also align with the importance attributed to interaction in online learning environments. This work suggests ways to encourage career behaviours in Higher Education and equip prospective labour market entrants with career resources.

## Introduction

It is not a novelty that entering the labour market represents a challenging developmental stage for young people, as is the case for new entrants from Higher Education. They go through identity and role transition and must handle tasks which could determine their later career development and success (Fouad and Bynner, 2008; Koen et al., 2012; Saks, 2018; Akkermans et al., 2021). Such a stage occurs in a graduate labour market scenario marked by discontinuity and uncertainty (Akkermans et al., 2015, 2021) or, as in the case of Italy, low capability to value graduates (OECD, 2017; Italian Government, 2021). The COVID-19 pandemic has even worsened this situation, downplaying new entrants’ career prospects and jeopardising a sustainable entrance into the world of work (Mahmud et al., 2021; Busetta et al., 2022).

As career self-management (henceforth, CSM) proactive behaviours are key to bearing the burdens of the transition (Saks, 2018; Haenggli et al., 2021), the present study delves into Career Engagement (henceforth, CE). CE is a unique general variable tapping the overt execution of several career preparation behaviours (Hirschi et al., 2014). The current literature has not fully discussed this general variable’s role in the CSM process, especially in generating career resources. Therefore, consistently with the Conservation of Resources Theory (COR)-based model of CSM (Hirschi, 2012; Hirschi et al., 2017), we attempt to analyse the functionality of CE, assuming that it develops Movement Capital, a group of career resources decisive for new entrants’ mobility during the transition stage (Forrier et al., 2009, 2015).

This study also intends to explore what motivates CE and the subsequent gain in career resources in new entrants from Higher Education. Literature acknowledges that Higher Education institutions are socially responsible for enhancing new entrants’ employment potential (Bridgstock and Jackson, 2019; López-Miguens et al., 2021). They are supposed to activate their Meso-level actors (e.g., teaching staff, career services, and work placement supervisors) to enhance students’ and graduates’ concern for their career development and empower their employability capitals (Tomlinson, 2017; Donald et al., 2021). In this vein, scholars have been trying to understand and better articulate the role of higher education in encouraging the accumulation of new entrants’ capital of career-related resources through CSM behaviours (e.g., Clarke, 2018; Clements and Kamau, 2018; Donald et al., 2021). Coherently, we aim to test the hypothesis that Support from Teaching Staff (henceforth STS)—a form of social support that university teachers can provide students with for their career development (Petruzziello et al., 2022)—influences CE behaviours and, in turn, leads to Movement Capital.

Lastly, the COVID-19 pandemic suddenly disrupted place-based classes globally in favour of remote learning as a contingency solution to prevent the spread of the contagion (UNESCO Institute for Statistics, 2022). Although most universities are returning to normal face-to-face learning, some institutions are trying to make use of the experience and competencies acquired during the pandemic emergency. Some online didactical activities are being experimented to become permanent in the post-pandemic period and make education more accessible (e.g., blended lectures; University of Bologna, 2022; University of Chieti, 2022). This issue requires understanding whether educational activities conducted in online spaces may influence the impact of supportive teaching on CSM-related factors. Thus, another goal of this study is to test whether online interaction between teachers and students is a moderating variable that influences the effect of STS on CE.

An added value of this study is expanding the literature on the resource-based view of CSM (Hirschi, 2012; Hirschi et al., 2017), incorporating CE as a crucial variable and corroborating the idea that investing in multiple behaviours may increase the necessary career resources to boost employment potential. CE allows assessing the simultaneous impact of a host of behaviours instead of analysing behaviours’ influence singly, as most research has done based on specific theories (e.g., Chiesa et al., 2020). In addition, studying whether and how STS affects CE and Movement capital may enrich the current understanding of contextual Meso-level factors facilitating the transition stage (Hirschi and Freund, 2014; Renn et al., 2014; Ma and Bennett, 2021; Okolie et al., 2022). The role of online interaction quality may provide further theoretical confirmations and new insights into theoretical models of online learning quality and evaluation (e.g., Peltier et al., 2007). In practical terms, our results may suggest how Universities – a prominent stakeholder in new entrants’ employment – can promote and facilitate a sustainable transition to the labour market.

## Hypotheses development

### Career engagement and movement capital

Self-directed and proactive behaviours help those entering the world of work to build a vantage position for negotiating with employment gatekeepers and achieving success (De Vos et al., 2009; Hirschi et al., 2014; Lo Presti et al., 2022). In this regard, Hirschi et al. (2014) introduced the concept of CE as “the degree to which somebody is proactively developing his or her career as expressed by diverse career behaviours” (p. 3). CE pertains to the exhibition of a range of behaviours: (a) career planning; (b) self-and environment exploration; (c) networking; (d) human capital development; (e) positioning behaviours. It is a higher-order variable reflecting how people are actively concerned about their careers without distinguishing between diverse career behaviours, coherently with individuals multiplying their efforts during transition stages (Hartung, 2013; Hirschi et al., 2014). Previous studies with new entrants have shown that CE promotes job and career satisfaction (Hirschi et al., 2013, 2014; Hirschi and Jaensch, 2015; Baluku et al., 2021).

Literature usually identifies career resources as driving forces of career behaviours (Lent and Brown, 2013; Šverko and Babarović, 2019). On the contrary, we draw upon the resource-based understanding of CSM (Hirschi, 2012) to explain the reverse relationship and, coherently, posit that CE influences Movement capital. Borrowing from COR theory (Halbesleben et al., 2014; Hobfoll et al., 2018), this resource-based approach asserts that CSM is a process in which people build and maintain pools (or “caravans”) of highly connected career resources through proactive behaviours. Movement capital^1^ is an example of a caravan of resources, as it taps personal factors facilitating career mobility and employability (Fugate et al., 2004; Forrier et al., 2015). Three main dimensions of Movement Capital are: Career Identity (CI), namely how individuals see and understand themselves as workers in a particular professional field (Fugate et al., 2004; Trede et al., 2012); Social Capital (SC), reflecting the network of relations people build through investing in formal and informal interpersonal relationships (Seibert et al., 2001); Personal Adaptability, herein represented—in line with Fugate et al. (2004) and González-Romá et al. (2018)—with the notion of Self-efficacy (SE), namely the individuals’ judgement about their ability to perform across different situations and overcome unexpected and adverse changes (Bandura, 1997).

Research has already found that typical career behaviours included in CE, such as self and environmental exploration and networking, are connected with components of movement capital, such as CI and SC (e.g., Hirschi, 2012; Praskova et al., 2015; Wolff and Spurk, 2020). Nevertheless, other behaviours encapsulated in CE may concur in forming specific career resources. Indeed, this study posits these behaviours to be studied together, as their combined execution is crucial for the transition to work (Hartung, 2013; Hirschi et al., 2014). This assumption aligns with the COR theory’s claim that people can multiply their investment (namely, perform several career behaviours) to enrich resource caravans for salient developmental stages (Hobfoll et al., 2018). Arguably, behaviours typically involved with forming specific resources (i.e., networking for SC, exploration for CI) facilitate access to other resources, and all behaviours included in CE may form CI, SC and SE. Past research has shown that investing in networking, planning, positioning and skills development is positive for having more precise self-and work-related information. Such information supports self-and environment-knowledge and interest development, resulting in a clearer identity (e.g., Meijers et al., 2013; Yuan et al., 2018). Also, exploration, planning, skills development, and positioning behaviours may lead to a serendipitous creation of social ties yielding better SC (e.g., Noda and Kim, 2014; Porter and Woo, 2015; Jackson, 2016a; Inceoglu et al., 2019). Exploration and planning for career goals (e.g., Hirschi, 2010; Cordeiro et al., 2015; Saks et al., 2015), networking (e.g., Nadermann and Eissenstat, 2018), skill development, and positioning (e.g., Jackson, 2016a; Inceoglu et al., 2019) are crucial for SE. Specifically, these are valued direct and indirect learning experiences nurturing confidence and faith in the capacity to use skills successfully in many career development tasks and challenges.

Still, the unique effect of a system of overt behaviours on career resources has received little attention, with Hirschi et al. (2014) being an exception in finding a positive relationship between university students’ CE with CI and SE.

### The predicting role of STS

#### STS and movement capital

Analysing the CE behaviours-based process of CSM must not discount the role of the context, also referred to as the ecosystem in which career progress occurs (Akkermans et al., 2021). As part of the graduate employment ecosystem, higher education institutions are responsible for supporting new entrants’ transition (López-Miguens et al., 2021). Such a task incorporates preparing and providing them with resources to support self-directed entrance at work (Bridgstock and Jackson, 2019), including career resources (Tomlinson, 2017; Clarke, 2018).

Scholarly work (e.g., Donald et al., 2018, 2021; Zhang et al., 2018, 2021; López-Miguens et al., 2021) detect such a contextual influence on transition to work in Meso-level factors. Meso-level factors are universities-related agencies—such as career services, teaching staff, academic support, alums, and graduate recruiters—supporting the transition from education to the labour market. From a COR theory perspective, this support form may reflect a resource caravan passageway, namely higher-order conditions (e.g., organisational support factors) that facilitate the creation or retention of resources (Akkermans et al., 2015; Hobfoll et al., 2018). López-Miguens et al. (2021) indicated teaching staff as one of the Meso-level factors. Recently, Petruzziello et al. (2022) advanced the concept and introduced STS, describing it as the amount of teachers’ social support that university students perceive from their teachers, which may enhance their career resources and employability. STS pertains to instrumental, informational, self-appraisal, and emotional support, provided by encouraging collaborative, work-based, career-oriented and emotional-supportive teaching to prepare students for the world of work beyond the classical lecture-based approach. Following the resource-based view of CSM (Hirschi, 2012; Hirschi et al., 2017), we assert that STS is a social resource stemming from a resourceful university environment acting as a passageway. Moreover, COR theory assumes that having resources at disposal may activate gain spirals to expand the resources’ caravan (Hobfoll et al., 2018). Then, we also posit that STS facilitates the accumulation of Movement Capital resources.

First, Scholarly research has acknowledged the role of higher education activities in developing students’ CI (Trede et al., 2012). According to Jackson (2016b), supportive teachers who encourage collaborative, problem-and work-based learning and career discussions define a community of practise in which students can socialise with and internalise the requirements and values of a professional role (Kuijpers et al., 2011; Trede et al., 2012; Tomlinson, 2017). In turn, they can commit to a clearer and more meaningful idea of whom they want to be as professionals (e.g., Cornelissen and van Wyk, 2008; Santisi et al., 2018).

Second, STS is expected to contribute positively to students’ SC. University students can access career-related information to nurture their professional social networks (Batistic and Tymon, 2017). Teachers define the structure through which students empower their network of contacts and relationships. Indeed, Kuijpers et al. (2011) found that teachers offering opportunities to experience real-life work problems and career-related discussions engage students to be concerned about their career future, fostering their networking capability. Donald et al. (2018) remarked that university teachers’ knowledge of the labour market might make information about useful contacts within workplaces and graduate job opportunities available to students. Therefore, it is arguable that STS represents a propeller of the students’ SC.

Third, SE is sensitive to learning practises where individuals can get involved in direct and indirect performance experiences (Bandura, 1997). Teachers can stimulate students’ SE by regularly encouraging them to work on problems from real work or explaining ways to master them with practical examples (e.g., Tan et al., 2016; Liu et al., 2020). Moreover, research has shown that promoting collaboration and confrontation may expose students to different ideas to get constructive feedback from peers on their solutions to real problems (e.g., Ehiyazaryan and Barraclough, 2009; Tan et al., 2016). This kind of learning can help students understand how they can harness knowledge and skills in the face of their future role demands, raising their efficacy beliefs (van Dinther et al., 2011). Also, research has shown that teachers interacting with students about their career future can be influential role models impacting students’ sense of efficacy about relevant transition aspects (e.g., decision-making; Zhang et al., 2018).

#### The mediation of CE between STS and movement capital

Herein, we posit that the mediating role of CE can explain the STS-Movement Capital relationships. Therefore, STS may initiate the CSM process of acquiring career resources through CE behaviours. In this regard, COR theory asserts that those who perceive social resources in a resourceful environment have higher chances of starting self-initiated behaviours for accumulating further resources to reach their career-and work-related goals (Hobfoll, 2002; Halbesleben et al., 2014; Hu et al., 2019). In this vein, Meso-level actors (i.e., support of significant others such as families, work placement supervisors, and peers) offer a solid foundation for career development efforts (Blustein, 2011). Indeed, they can provide career-related information, guidance for transition tasks, development and emotional support. This may make students concerned with their future and confident in engaging in proactive behaviours, such as exploration, planning, and skills development (e.g., Buhl et al., 2018; Jiang et al., 2019; Ma et al., 2022; Okolie et al., 2022).

Drawing upon these foundations, we argue that STS reflects environmental conditions that may elicit students’ CE. Previous scholarly work with new entrants hints at this relationship. Kuijpers and Meijers (2012) and Kuijpers et al. (2011) suggest that STS forms of support encourage students to be concerned with their careers and invest in CE behaviours. Teachers who create conditions for collaborative and work-based learning and discussions about careers may provide students with information to perform self-exploration of their values, interests, and strengths (Kuijpers and Meijers, 2012; Dickerson et al., 2016) or with insights into the actual world of work, therefore eliciting environmental exploration and networking (Kuijpers et al., 2011; Kuijpers and Meijers, 2012; Donald et al., 2018). Work-and career-based learning may help students to disclose wishes and motives and inform students’ career-related decision-making and planning (Zhang et al., 2018). Supporting students to get familiar with the reality of work environments can help them to understand work requirements and career possibilities (i.e., Ehiyazaryan and Barraclough, 2009; Kuijpers and Meijers, 2012; Jackson, 2016a), thus stimulating skills development and positioning behaviours (Kuijpers and Meijers, 2012). Hirschi and Freund (2014) detected a positive impact of social career support (encapsulating family, peers, and teachers’ support) on CE among German students. Yet, the role of teachers has not been addressed in detail as much as the STS concept can. This study attempts to fill this literature gap and extend the understanding of the University Meso-level factors, specifically teaching staff, in facilitating the CSM process (i.e., CE to Movement capital).

Therefore, we put together the previously discussed relationships between STS, CE and Movement capital and contend that STS predicts the CSM process leading to Movement Capital, arguing that CE acts as a bridge between STS and Movement capital. Therefore, we propose the following:

*Hypothesis 1*: CE mediates the relationship between STS and Movement Capital.

#### The moderating role of online interaction

During the COVID-19 pandemic, a shift to online learning was observed in many countries where modern information technology was used to deliver the learning contents initially planned for the classroom setting (Zhao et al., 2022). Despite institutions’ efforts to ensure the continuity of education through online classes, the COVID-19 pandemic bore an academic impact on students, who were reported to be less motivated in learning and were experiencing dilemmas about the future of their careers (Nell et al., 2020). Furthermore, Jemini-Gashi and Kadriu (2022) provided intriguing insights regarding the STS that Kosovo students received in the online learning context. The students regarded online learning as unsuitable for providing psychological, social, or professional support for career guidance and decision-making (Jemini-Gashi and Kadriu, 2022). They specifically commented on their teachers’ lack of career orientation support. The context of the COVID-19 pandemic thrust the world into the first large-scale online learning experiment (Zhao et al., 2022), with no prior conception of how the interaction in the online learning context should be carried out. The qualitative results of Price et al. (2007) study indicated that students’ and their tutors’ conceptions of their roles in an online environment need to be addressed to achieve effective online communication and interaction. Differences in conceptions or a lack of clarity regarding their roles in an online learning context may have affected the experiences of online interaction between students and teachers during the COVID-19 pandemic. In general, the major models of technology acceptance, use and success applied to learning systems acknowledge the teacher-student interaction (e.g., responsiveness, timeliness of feedback, communication, and social closeness) as an important factor influencing online learning experiences (Paechter and Maier, 2010; Kang and Im, 2013; Al-Fraihat et al., 2020). Indeed, as Bennett et al. (2020) pointed out, the learning spaces modulate teaching modes, thus moderating the influence of teaching strategies on learning process outputs.

Therefore, it is possible to argue that, on the one hand, the online learning context presents challenges to an effective learning experience because of the lack of immediateness of physical closeness in terms of human contact and chances of direct exchange and discussions (Dumford and Miller, 2018). On the other hand, positive teacher-student interaction can be observed in the online learning context through the efficient provision of means for interaction and feedback from the instructor, and it can be beneficial for learning effectiveness (Paechter and Maier, 2010). Therefore, we translate these assumptions in the context of our study to argue that if online interaction represents a crucial factor in the effectiveness of online learning experiences, it may also imply that it has repercussions on how the learning environment at university affects the CSM process. For instance, Heymann et al. (2022) theorised that teachers are major actors and support providers to promote students’ employability while learning is implemented through online platforms. Based on what was reported above, one may think that higher levels of online interaction may positively moderate, and boost, the STS-CE relationship. However, Dumford and Miller (2018) found that online learning is an obstacle to collaborative learning, which is a core facet of STS. Therefore, we adopt an exploratory approach to better understand the effect of online learning and online interaction, taking into account the relationships hypothesised above. In other words, we attempt to understand the potential and the direction of the moderating effect of online interaction in the relationship between STS and CE, which may affect the indirect effect of STS on the Movement Capital through CE. We formulate the following:

*Research Question 1*: Does online interaction between teachers and students moderate the relationship between STS and CE so that the indirect effect of STS on Movement capital is different at varying levels of online interaction?

## Materials and methods

### Procedure and participants

The procedure for data collection, which started in March 2020, adhered to the Ethical Guidelines of the Helsinki Declaration (World Medical Association, 2013) and the Italian Association of Psychology (2015). It was authorised by the ethical committee of the Authors’ institution. In line with the study’s rationale, we recruited participants with a purposive sampling strategy. Therefore, the selection criterion was to be enrolled in a degree course (bachelor’s or master’s). We spread the invitation to participate *via* social networks (e.g., Facebook, LinkedIn). The invitation contained a hyperlink to take the survey on the online platform Qualtrics. The survey’s introduction explained the rationale, the goal, and the characteristics of the study, assuring anonymity and confidentiality in the data treatment process in line with EU regulation no. 679/2016. Participants could then give their informed consent to participate voluntarily, with the right to withdraw at any time. A total of 535 people interacted with the survey. After removing cases with many missing values (more than 30%) and dropping cases that did not match the selection criteria, 267 resulted as valid cases (response rate of 49.91%), mostly Women (86.1%; Man = 13.1%; Non-binary = 0.7%), with a mean age of 25.63 years (*SD* = 5.84). Most participants already had work experience (76.8%) compared to those without experience (23.2%) and were enrolled in a Humanities degree course (78.7%) compared to those in the Scientific (20.2%) and Health (1.1%) fields.

### Measures

#### CE

We used the Italian version of the Career Engagement scale (Hirschi et al., 2014), adapted in Italian by Petruzziello et al. (2021). The scale consisted of nine items (e.g., “To what extent have you in the past two months…” “…actively sought to design your professional future?”) assessed with a Likert response scale ranging from 1 = *almost never* to 5 = *very often*. Excellent internal consistency was found (Cronbach’s alpha value = 0.89).

#### Movement capital

The measure developed by González-Romá et al. (2018) was used for participants’ Movement Capital by assessing CI (four items), SC (two items), and SE (three items). The scale employed a Likert response scale ranging from 1 = *completely disagree* to 5 = *completely agree* to assess the items (e.g., “I strongly identify with my chosen line of work/career field”). We averaged these items to create an overall score for movement capital, with good internal consistency (Cronbach’s alpha value = 0.83).

#### STS

We used the 19-item scale developed by Petruzziello et al. (2022). The items assessed the various facets of STS. The scale assesses support for collaborative and work-based learning (13 items, e.g., “When solving a work problem, we are encouraged to draw on our existing knowledge”; “We are encouraged to discuss with fellow students how we study the subject matters”). Also, support in discussing career information and opportunities is measured (two items, e.g., “If I needed it, I could ask my teachers to discuss my professional future”). Emotional support is also evaluated (four items, e.g., “We can rely on the teachers in this programme”). Participants had to rate their agreement with the items on a Likert response scale ranging from 1 = *not at all* to 5 = *completely*. We averaged these items to create an overall score for STS, with a good internal consistency (Cronbach’s alpha value = 0.92).

#### Online interaction

We assessed online interaction between teachers and students with four items by Paechter and Maier (2010), evaluating teachers’ social support to facilitate students’ learning in online didactical activities. The items (e.g., “When I need advice from my tutor I can easily get in contact with her/him *via* e-mail, chat, forum etc.”) presented a five-point Likert response scale ranging from 1 = *completely disagree* to 5 = *completely agree*. We found good internal consistency (Cronbach’s alpha value = 0.83).

#### Control variables

We used participants’ age, gender, previous work experience, and study field as control variables. These variables are deemed to influence the CSM process in terms of modulating personal career resources or facilitating/inhibiting the process of resource accumulation (Hirschi and Koen, 2021).

### Strategy of analyses

Given the nature of the data collection, with self-report measures and a cross-sectional design, common method bias could affect the solidity of the analyses’ results (Podsakoff et al., 2003). Therefore, we performed the Harman’s one-factor test to evaluate the severity of this bias. Moreover, we ran a confirmatory factor analysis (CFA) with the AMOS software to test different measurement models for each variable. Cronbach’s alpha, Composite Reliability (CR), and Average Variance Extracted (AVE) also evaluated the internal consistency and convergent validity of the scales used in the study (Hair et al., 2019). The Fornell-Larcker criterion (Fornell and Larcker, 1981) was used to evaluate discriminant validity among all the study variables, which exists if the square root of the AVE of each variable is greater than the correlation coefficients between that variable and other variables.

We computed means, standard deviations, and bivariate correlations for each study variable. For the hypothesis and research question test, we used the IBM SPSS software, version 25. We employed the analytical approach and the macro PROCESS developed by Hayes (2018) to test the mediation hypothesis with Model 4 and the moderated mediating model for the research question with model 7. We used 5,000 bootstrapped samples with a 95% confidence interval for the models’ testing and effects estimation.

## Results

### Preliminary results

Harman’s single-factor test confirmed the low potential impact of common method bias since the single factor explained only 24.13% of the variance. To evaluate the goodness of fit of the measurement models, we used the Root Mean Square Error of Approximation (RMSEA), the Standardised Root Mean Square Residual (SRMR), the Comparative Fit Index (CFI), the Non-Normed Fit Index (NNFI), and the ratio of the model chi-square and the degrees of freedom (χ^2^/df). Values for CFI and NNFI at least ≥ 0.90, RMSEA and SRMR at least ≤ 0.08, and χ^2^/df < 3 suggest acceptable fit (Hair et al., 2019). Model fit was acceptable for each scale (see Table 1). Moreover, all the scales reported Cronbach’s alpha and CR (see Table 1) values above the acceptable threshold of 0.70 (Nunnally and Bernstein, 1994). AVE values were above the threshold of 0.50, except for CE, which showed AVE values close to the threshold (see Table 1). Fornell and Larcker (1981) asserted that if CR is at the recommended level, the convergent validity is still supported even in the presence of an AVE < 0.50. Lastly, the analyses met the requirements of the Fornell-Larcker criterion (Fornell and Larcker, 1981), providing evidence for the adequate discriminant validity of the scales (see Table 2).

**Table 1:** CFAs’ goodness of fit and measures’ validity convergent and discriminant validity.

Variable	CFA models’ goodness of fit					Measure’s validity		
	χ^2^/df	CFI	NNFI	RMSEA	SRMR	α	CR	AVE
STS	2.23	0.933	0.921	0.068	0.079	0.92	0.96	0.76
ION	1.29	0.999	0.997	0.026	0.013	0.83	0.83	0.56
CE	2.65	0.969	0.952	0.079	0.039	0.89	0.89	0.49
MC	1.15	0.997	0.995	0.023	0.026	0.83	0.93	0.62

*N* = 216; STS, Support from teaching staff; ION, interaction online; CE, Career Engagement; MC, Movement Capital. χ^2^/df, ratio of the model chi-square and the degrees of freedom; CFI, Comparative Fit Index; NNFI, Non-Normed Fit Index; RMSEA, Root Mean Square Error of Approximation; SRMR, Standardised Root Mean Square Residual; α, Cronbach’s Alpha; CR, Composite Reliability; AVE, Average Variance Extracted.

**Table 2:** Correlations, means, and standard deviations.

	M	SD								
1. Age	25.63	5.84								
2. Gender^a^	-	-	0.12^*^							
3. Past work experience^b^	-	-	−0.11	−0.11						
4. Field of study^c^	-	-	0.03	−0.11	0.08					
5. STS	3.14	0.69	−0.01	−0.09	−0.04	0.02	**0.76**			
6. ION	3.59	0.79	−0.01	−0.04	0.02	−0.12	0.58^**^	**0.75**		
7. CE	3.27	0.90	0.003	0.11	−0.21^**^	−0.06	0.17^**^	0.15^*^	**0.71**	
8. MC	3.43	0.58	−0.08	−0.02	−0.14^*^	−0.01	0.42^**^	0.29^**^	0.59^**^	**0.79**

*N* = 267. STS, Support from teaching staff; ION, interaction online; CE, Career Engagement; MC, Movement Capital. Figures in italicised bold are the square root of each variable’s AVE (Fornell-Larcker criterion; Fornell and Larcker, 1981).

a 1 = Man; 2 = Woman; 3 = non binary.

b 1 = yes; 2 = no.

c 1 = Humanities; 2 = Sciences; 3 = Health.

* *p* < 0.05;

** *p* < 0.01.

### Test of the hypothesis and research question

**Figure 1:**
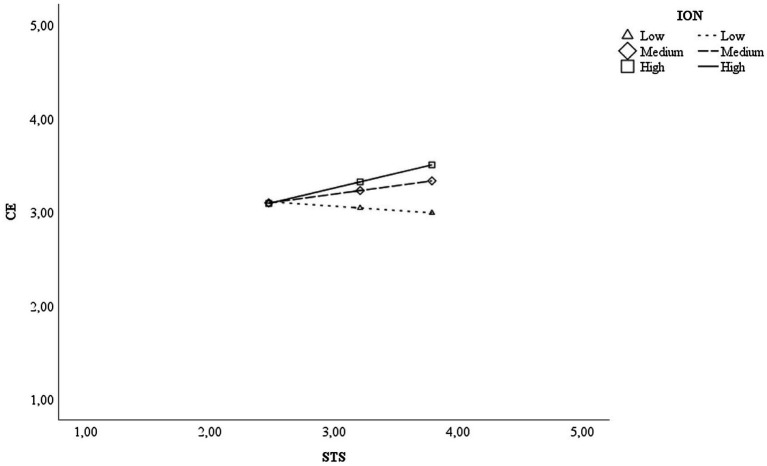
Interactive effect of STS and ION on CE. STS, support from teaching staff; ION, interaction online; CE, career engagement.

Table 3 shows the effects’ coefficients of the first model tested (H1). Results showed that STS positively affects CE, which, in turn, has a positive relationship with Movement Capital. The indirect effect results corroborate that STS impacts Movement Capital indirectly thanks to the mediation of CE (B = 0.08, CI [0.02; 0.13]), supporting Hypothesis 1. Perceiving support from teachers stimulates students’ CSM behaviours, triggering, in turn, the accumulation of crucial career resources. Concerning the moderated mediation model (see Figure 1; Table 4), we find a significant positive interaction effect between STS and Online interaction (B = 0.27 *p* < 0.001). Moreover, the conditional indirect effect analysis (Table 4) shows that the positive indirect effect between STS and Movement capital is significant and stronger at medium and high levels of Online Interaction.

**Table 3:** Summary of the Mediation Analysis (Hypothesis 1).

Variable	Mediator (CE)			Dependent variable (MC)				
Control variable	B	Standard error	*t*-value	*p-*value	B	Standard error	*t*-value	*p-*value
Age	−0.004	0.001	−0.45	0.65	−0.01	0.00	−1.70	0.09
Gender^a^	0.25	0.16	1.64	0.10	−0.06	0.08	−0.83	0.41
Past work experience^b^	−0.41	0.13	−3.19	0.00	−0.04	0.06	−0.69	0.49
Field of study^c^	−0.07	0.12	−0.58	0.56	0.02	0.06	0.41	0.68
**Independent variable**								
STS	0.21	0.08	2.79	0.01	0.27	0.04	6.94	0.000
CE					0.35	0.03	11.31	0.000
*R^2^*	0.08				0.46			
*F*	4.58				37.32			
Indirect effect					B	Standard error	LL	UL
STS → CE → MC					0.08	0.03	0.02	0.13

*N* = 267; STS, Support from teaching staff; ION, interaction online; CE, Career Engagement; MC, Movement Capital.

a 1 = Man; 2 = Woman; 3 = non binary.

b 1 = yes; 2 = no.

c 1 = Humanities; 2 = Sciences; 3 = Health.

**Table 4:** Summary of the moderated mediation analysis (Research Question 1).

Variable	Mediator (CE)				Dependent variable (MC)			
Control variable	B	Standard error	*t*-value	*p-*value	B	Standard error	*t*-value	*p-*value
Age	−0.01	0.01	−0.59	0.56	−0.01	0.00	−1.70	0.09
Gender^a^	0.28	0.15	1.85	0.07	−0.06	0.08	−0.83	0.41
Past work experience^b^	−0.41	0.13	−3.33	0.001	−0.04	0.06	−0.69	0.49
Field of study^c^	−0.02	0.12	−0.13	0.89	0.02	0.06	0.41	0.68
**Independent variable**								
STS	−0.83	0.30	−2.76	0.01	0.27	0.04	6.94	0.000
ION	−0.68	0.24	−2.79	0.01				
STS × ION	0.27	0.08	3.42	0.001				
CE					0.34	0.03	11.32	0.000
*R^2^*	0.13				0.46			
*F*	5.29				37.32			
Index of moderated mediation	Index	Standard error	LL	UL				
	0.09	0.03	0.04	0.15				
Conditional indirect effects	B	Standard error	LL	UL				
Low IS	−0.03	0.04	−0.11	0.05				
Medium IS	0.06	0.03	0.002	0.13				
High IS	0.11	0.04	0.04	0.18				

*N* = 267; STS, Support from teaching staff; ION, interaction online; CE, Career Engagement; MC, Movement Capital.

a 1 = Man; 2 = Woman; 3 = non-binary.

b 1 = yes; 2 = no.

c 1 = Humanities; 2 = Sciences; 3 = Health.

## Discussion

Career behaviours are essential to counteract the complexity of entering a contemporary employment environment and increase one’s chances of going through an adaptive and sustainable transition stage (Akkermans et al., 2021). Accordingly, we aimed to delve into the CE concept and investigate its impact on Movement Capital’s development. Moreover, we aimed to articulate better the role of the university-related Meso-level factors in encouraging career behaviours in the CSM process and posited that STS could influence CE behaviours.

Adopting the resource-based view of CSM (Hirschi, 2012; Hirschi et al., 2017), we posited that the relationships between STS, CE, and Movement Capital define a process of career resource acquisition. Accordingly, we tested a mediation model hypothesising CE as a crucial explanation of the connection between STS and Movement capital (Hypothesis 1). We further examined the relationship between STS and CE by exploring the moderating effect of online interaction in the said relationship (Research Question 1). We tested a mediation and a moderated mediation model with data obtained involving a sample of Italian university students. As expected, STS had a significant and positive effect on CE, and CE had a significant and positive effect on Movement Capital. In support of our hypothesis, CE mediated between STS and Movement Capital. Our findings are consistent with the resource-based view of CSM and align with previous evidence of a relationship between career behaviours and career resources (e.g., Meijers et al., 2013; Porter and Woo, 2015). In response to our research question, the moderated mediation showed medium and high levels of online interaction moderating the STS-CE relationship and the hypothesised indirect effect. In other words, the indirect association between STS and Movement Capital is stronger at medium and high levels of STS. However, the effect of STS on CE is nonsignificant at low levels of online interaction, further remarking on the importance of high-quality online interaction.

### Theoretical and research implications

Our study enriches early career literature in some ways. First, the positive relationship between CE and Movement Capital is consistent with the resource-based understanding of CSM as the building and maintenance of caravans of career resources go through proactive behaviours (Hirschi, 2012; Hirschi et al., 2017). More than this, in testing the role of CE on Movement capital, this study goes beyond the widely recognised and used CSM view, which sees career resources as motivational drivers of career behaviours (e.g., Lent and Brown, 2013; Šverko and Babarović, 2019). Corroborating that this relationship works the other way around valuably adds to the study of CSM, suggesting research to consider the relationship’s bidirectional nature in unravelling the complexity of CSM (Hirschi and Koen, 2021). Moreover, our study has the merit of examining CSM behaviours as a collective system of overt behaviours (i.e., CE). It confirms that the co-occurrence of career behaviours plays a crucial role in the starting stage of one’s career. In addition to its alignment with the COR theory-related notion that people tend to increase their efforts during the developmental stage (Hobfoll et al., 2018), this finding concurs with recent theoretical development in CSM studies (Hirschi and Koen, 2021) and recent literature in the early career field (Ma and Bennett, 2021). Therefore, we argue that people confronted with the entrance into the world of work should equally devote their efforts to diverse behaviours to accumulate resources and facilitate their advancement. Future research is advocated to account for multiple behaviours to understand and explain career resources accumulation, transition-to-work performance and success.

Second, support for the predicting role of STS parallels the findings that support from significant others encourages concerns for career construction among young people, eliciting career behaviours (e.g., Buhl et al., 2018; Ma et al., 2022; Okolie et al., 2022). Our findings further progress this assumption with a theoretical value. Indeed, different from research that has stressed support for career development at a general level (e.g., Hirschi and Freund, 2014), our study focused specifically on supportive teaching at university, adopting a comprehensive conceptualisation encapsulating different forms of such support. This is in line with the need to delve into teaching support more in-depth, as suggested recently in early career research (Zhang et al., 2021). In this way, we also responded to the call to better articulate how higher education facilitates the entrance to the world of work (Clarke, 2018), reinforcing the notion of teaching staff as a crucial Meso-level actor in higher education for career development (López-Miguens et al., 2021; Petruzziello et al., 2022). As STS is a driver of career behaviours in new entrants, this study also corroborates the notion that agency in career management may have, in the context, a decisive facilitator (Akkermans et al., 2021; Hirschi and Koen, 2021). We advocate future research incorporating STS’ role in understanding career development at early stages.

Third, our results provide interesting insights concerning the online learning context. It is noteworthy that STS is still an important social resource for students to engage in CSM behaviours when situated in an online learning environment. However, our results suggest that medium to high levels of online interaction should be maintained between student and teacher for the teacher’s support to effectively encourage students to perform CE behaviours. Such findings confirm that student-teacher interaction in the online learning environment is crucial to determine learning achievements, supporting the theorising proposed by the major models of technology acceptance, use, and success applied to learning systems (Paechter and Maier, 2010; Al-Fraihat et al., 2020). Recent studies have suggested that students appreciate increased online teacher-student interaction because they receive more clarity regarding course content and information (e.g., Mukhtar et al., 2020). Though past studies have noted the importance of instructor involvement and interaction in distance learning (Zhao et al., 2005), our results contribute to the literature by looking beyond students’ academic outcomes. Rather, we highlight that satisfactory levels of online interaction allow teachers to continue their supportive roles in developing students’ concerns towards their career future and promoting their CSM behaviours despite the absence of a physical learning environment. Future research on the implications of adopting online learning systems in understanding the role of teaching on students’ career development may better articulate online interaction by examining the sub-factors of learner–instructor interaction as theorised by Kang and Im (2013). Also, replicating this study’s model by including other influential factors in online learning (e.g., type of online platform or interface, educational system quality, learner characteristics; Al-Fraihat et al., 2020) can enrich the understanding of online teacher-student interactions’ role in promoting students’ career behaviours.

### Practical implications

The findings highlight universities’ role, especially teachers, in fostering students’ CE behaviours and movement capital. Our study has implications for the university’s function to promote CSM behaviours of students, especially through the support and development opportunities they provide their teachers with. One implication is for universities to give more attention to teachers’ training and development to broaden their pedagogic practises beyond the achievement of academic learning outcomes. Specifically, more work-and career-based learning strategies can be integrated into the teachers’ course syllabus or curricular activities to promote students’ career development. Universities or teachers can seek partnerships or collaborations with employment agencies, career services, university alums, and industrial organisations to have the necessary teaching resources and information to stimulate students’ insights and discussions on their career decision-making and career planning (Akkermans et al., 2015). In general, promoting proactive career behaviours among students would entail adapting curricular course activities that allow students to increase their movement capital. For example, to expand their network and improve their SC, experts or speakers from relevant career fields can be invited to share advice with students to supplement the teacher’s knowledge of the field (López-Miguens et al., 2021). Another example is incorporating in-class workshops or seminars on competencies development to let students practise their skills and perform tasks that enhance their SE (Liu et al., 2020). Lastly, collaborative exercises that simulate work situations can help students better understand themselves as future workers, thus allowing them to define and reinforce their CI.

In light of the events during the COVID-19 pandemic that positioned teachers to use unaccustomed technology for their teaching methods, our study also highlights the importance of providing support to boost teachers’ technological competencies. Concerning our findings, ensuring better online interaction between teachers and students entails more preparation on teachers’ ends to achieve a smoother flow of course delivery (Dumford and Miller, 2018; Kulikowski et al., 2021). Providing sufficient technological assistance and guidance to teachers will benefit them in their course preparations and presentations while addressing the logistical challenges of online learning. To help students not accustomed to the online learning environment, our study advises monitoring online interaction levels in which teachers can encourage two-way feedback and communication with the students. Achieving satisfactory levels of online interaction can help students stay engaged in self-regulated learning and apply career behaviours that prepare them to transition to work.

### Limitations and future research recommendations

Some limitations of the study need to be considered. First, the study employed a cross-sectional design, which makes it difficult to draw conclusions about causality. Future research can focus on the longitudinal relationships of the variables discussed in this study. Such research can also assess the trajectories of STS and CE over time and their longitudinal impact on students’ Movement Capital, in line with COR theory recommendations of analysing fluctuations in resource investment and resource gain spirals in different environmental conditions and through life stages (Halbesleben et al., 2014). Second, the study is based on self-reported measures. Though our study tested for the low potential impact of common method bias, future research can benefit from using other data sources, such as teachers’ perceptions or evaluations of their support functions concerning students’ CE behaviours (Parola et al., 2022). This may align with existing research, which has already shown robust evidence reporting positive relationships between the self-reported provision of support and the career development of support receivers (e.g., parents’ self-reported supportive behaviours and their children’s career development, Jiang et al., 2019; Liang et al., 2022). Third, our sample was not characteristic of the general student population in the Italian university context, given the dominance of women and Humanistic students participating in this study. Similar studies in the future should opt to use more representative samples to increase the generalisability of the results. Fourth, our study used a measure of online interaction that was developed before the events of the COVID-19 pandemic and its impact on distance learning. Future research can add or use items that measure the more recent experiences of teacher-student interaction in the online learning environment.

## Data availability statement

The raw data supporting the conclusions of this article will be made available by the authors without undue reservation.

## Ethics statement

Ethical approval was provided by the Bio-ethical Committee of the University of Bologna. The participants provided their written informed consent to participate in this study.

## Author contributions

GP: conceptualisation, methodology, formal analysis, investigation, data curation, writing—original draft, and visualisation. AA: data curation, writing—original draft, and visualisation. RC and MM: supervision and project administration. All authors contributed to the article and approved the submitted version.

## Conflict of interest

The authors declare that the research was conducted in the absence of any commercial or financial relationships that could be construed as a potential conflict of interest.

## Publisher’s note

All claims expressed in this article are solely those of the authors and do not necessarily represent those of their affiliated organizations, or those of the publisher, the editors and the reviewers. Any product that may be evaluated in this article, or claim that may be made by its manufacturer, is not guaranteed or endorsed by the publisher.

## Footnotes

^1^Human capital, which is considered part of Movement capital (Forrier et al., 2015), generally refers to an individual’s level of education and skills gained outside of the university setting (Fugate et al., 2004). It was not considered in this study as it goes beyond the scope of examining students’ motivation for CSM behaviours nurtured within the university classroom context and the subsequent gain in career resources. Social capital, however, can be considered a human capital-related factor in labour market entrants (see Donald et al., 2019).
